# Eosinophils, beyond IL-5

**DOI:** 10.3390/cells10102615

**Published:** 2021-10-01

**Authors:** Stephane Esnault, Mats W Johansson, Sameer K Mathur

**Affiliations:** 1 Department of Medicine, Division of Allergy, Pulmonary and Critical Care Medicine, School of Medicine and Public Health, University of Wisconsin-Madison, Madison, WI 53792, USA; 2 Metabolism Theme, Morgridge Institute for Research, Madison, WI 53706, USA; 3 Department of Biomolecular Chemistry, University of Wisconsin, Madison, WI 53706, USA

New therapeutic monoclonal antibodies targeting the IL-5/IL-5 receptor pathway are extremely efficient in depleting blood eosinophils from subjects with asthma. In asthma, these anti-IL-5 therapies reduce exacerbations by 50% in eosinophilic severe asthma, but they are not available for patients with milder eosinophilic asthma. In addition, it is well known that these therapies do not totally deplete lung eosinophils and do not seem to change their phenotype [1,2]. Eosinophils are produced in IL-5 knock-out mice, and patients receiving neutralizing anti-IL-5 therapies retain a stable population of residual blood eosinophils closely similar to those of healthy individuals [3,4]. In fact, it is uncertain whether the lack of complete depletion of either residual or activated eosinophils is beneficial or detrimental for patients. Despite the advantage of the anti-IL-5 specificity on eosinophils, the redundancy of the three beta chain receptor cytokines (IL-3, IL-5 and GM-CSF) on eosinophils is well known and may explain the lack of complete depletion of eosinophils [5]. However, in mouse, blockade of the common beta chain receptor did not completely deplete the presence of terminally differentiated eosinophils in the blood [6], suggesting that other factors can differentiate and mature eosinophils. Of note, asthma is only one of numerous eosinophilic diseases, and anti-IL-5 therapies have not yet proven their clinical efficacy to treat most of these devastating diseases. In this Special Issue, several original studies and reviews point out the necessity to continue our efforts to better characterize the heterogeneity of precursor and mature eosinophil populations and to provide new insights regarding other therapeutic targets, which would act on the differentiation and activation of general and specific eosinophil populations.

Among the original studies, Coden et al. [7] report the development of a new protocol to differentiate a functional and long-lived tissue-resident type of eosinophils using IL-5-independent steps. These steps require the presence of stromal cells that develop during eosinophil differentiation. While the stromal cell mediators leading to this eosinophil phenotype and their metabolic reprogramming remain unknown, the authors discuss tenascin-C and GM-CSF as possible candidates to explain these changes. 

Polarization and migration are critical steps for eosinophils to be recruited from the blood to tissues and to navigate in tissues. Related to this subject, Son et al. [8] describe that mechanical stress leads to eosinophil flattening and membrane protrusions, which are both important events during eosinophil extravasation. They demonstrate mechanisms and cause–effect relationships between fluid shear stress (mechanical stress)-activated eosinophils, intracellular calcium release and cytoskeleton reorganization during cell migration using state-of-the-art real-time confocal microscopy and pharmacological inhibitors. Their data call attention to new extra- and intracellular mechanisms leading to eosinophil trafficking and accumulation into tissues. In the same topic, Shen et al. [9] identify the peptidyl-prolyl cis–trans isomerase, Pin-1, as an inducer of cytoskeletal re-organization, eosinophil morphology change and cell migration through the modulation of Rho GTPase activity. While IL-5 was used to activate Pin-1 in this article, both GM-CSF and IL-3, as well as matrix proteins such as hyaluronic acid, are known activators of Pin-1 in eosinophils [10,11]. 

The main effective functions of eosinophils are preceded by release of their intracellular factors by piecemeal release, exocytosis (degranulation) and cytolysis. The degranulation consequences on microbes, cells and tissues are potentiated by release of DNA (extracellular trap). Germic et al. [12] demonstrate that eosinophil degranulation is not concomitant with release of DNA, which occurs later after degranulation. In that study, degranulation was triggered by IL-5, GM-CSF or IFNG priming followed by activation with either complement factor 5 or eotaxin. In another study, Bernau et al. [13] use IL-3-primed eosinophils followed by an interaction with complexed IgG to reveal that products from degranulated and lysed eosinophils activate pulmonary fibroblasts to produce IL-6 and IL-8 in an IL-1-dependent manner. 

In addition to stromal cells, mechanical stress, immune soluble factors and extracellular matrix proteins, Tiwary et al. [14] show evidence that eosinophil exposure to virus triggers their activation. They found that eosinophil survival and adhesion molecules were dynamically regulated when exposed to influenza A virus. Influenza-activated eosinophils migrated out of the lungs efficiently to lymphoid organs and also participated in improving epithelial barrier responses to mitigate influenza pathogenesis. Finally, Koenderman et al. [15] describe that patients with COVID-19 display eosinopenia as well as blood eosinophils with refractory microbe-associated molecular pattern peptide (formyl peptide) activation. This study suggests that formyl peptide-sensitive blood eosinophils are recruited to the tissue in a non-T2 viral/microbial environment during infection.

Taken together, these original articles demonstrate the existence of multiple different eosinophil differentiators and activators as well as the heterogeneity of the eosinophilic response depending on the type of activator and the environment.

In one of the review articles, Salter et al. [16] discuss new potential drug targets to reduce eosinophilopoesis and eosinophil recruitment to airway tissues. They detail the intracellular molecular mechanisms and factors that lead to the development of eosinophil progenitors and mature eosinophils. Non-IL-5 factors include the other β-chain cytokines IL-3 and GM-CSF, CCR3 and the epithelial-derived alarmin cytokines IL-33, TSLP and IL-25. They also comment on evidence of eosinophilopoeisis occurring locally in airway tissue. In the same vein, Cusack et al. [17] detail the therapies that are used or are evaluated for treatment of eosinophilic asthma, including corticosteroids, the IL-3/5/GM-CSF axis, CCR3, type-2 cytokines and CRTH2, as well as Siglec-8, which is the focus of the article by Youngblood et al. [18]. That article relates to the history leading to the development of the humanized therapeutic antibody directed against Siglec-8 to deplete eosinophils. Siglec-8 is exclusively produced on the cell surface of eosinophils, mast cells and basophils, and the binding of the antibody leads to eosinophil death, e.g., by antibody-dependent cellular cytotoxicity (ADCC), in a caspase-dependent manner, as well as in cytokine-primed eosinophils. The therapeutic antibody is now in phase 2/3 clinical development for multiple eosinophilic diseases. 

Although eosinophils have some negative effects on numerous gastrointestinal diseases, in their review, Masterson et al. [19] stress the danger of depleting populations of eosinophils with beneficial anti-inflammatory and homeostatic functions, particularly in the intestine. They provide a remarkable detailed description of the eosinophil varieties (endotypes) regarding responses to in vivo environments and their numerous functions in tissues. Finally, Lype et al. [20] emphasize the unappreciated importance of basophils in the late phase of the allergic reaction and their influence on eosinophils. The article reviews the literature on the interplay between eosinophils and basophils in type-2 immunity in different tissues. Basophils act on eosinophil recruitment by producing IL-4. Furthermore, the authors discuss the advantage of targeting the IL-3/5/GM-CSF axis, particularly a product from basophils, IL-3, which is a major activator of basophils, eosinophils and plasmacytoid dendritic cells.

Thus far, only asthma patients with severe eosinophilic asthma receive treatment with anti-IL-5 pathway therapies. The efficacy in a few additional eosinophilic diseases has been established, such as eosinophilic granulomatosis with polyangiitis and hypereosinophilic syndrome. Their efficacy on numerous other eosinophilic diseases is under investigation. It remains to be seen whether approaches of blocking eosinophil activities that may be shared with other inflammatory cells is a more effective approach to manage eosinophil-associated diseases. Research should continue on alternative therapeutics to more specifically control eosinophil populations or activities that contribute to disease versus eosinophils with protective and homeostatic functions. Unlike other types of immune cells (i.e., lymphocytes and antigen-presenting cells), for which our knowledge is more advanced regarding their heterogeneity, many basic questions remain regarding the heterogeneity of eosinophils and specific mechanisms that lead to their damaging functions. 

## References

[1] Kelly E.A., Esnault S., Liu L.Y., Evans M.D., Johansson M.W., Mathur S., Mosher D.F., Denlinger L.C., Jarjour N.N. (2017). Mepolizumab attenuates airway eosinophil numbers, but not their functional phenotype, in asthma. Am. J. Respir. Crit. Care Med..

[2] Johansson M.W., Gunderson K.A., Kelly E.A., Denlinger L.C., Jarjour N.N., Mosher D.F. (2013). Anti-IL-5 attenuates activation and surface density of β_2_-integrins on circulating eosinophils after segmental antigen challenge. Clin. Exp. Allergy.

[3] Van Hulst G., Jorssen J., Jacobs N., Henket M., Louis R., Schleich F., Bureau F., Desmet C.J. (2021). Anti-IL-5 mepolizumab minimally influences residual blood eosinophils in severe asthma. Eur. Respir. J..

[4] Hassani M., Tak T., van Aalst C., van Nederveen S., Tesselaar K., Vrisekoop N., Koenderman L. (2021). Differential effects of short- and long-term treatment with mepolizumab on eosinophil kinetics in blood and sputum in eosinophilic asthma. iScience.

[5] Esnault S., Kelly E.A. (2016). Essential mechanisms of differential activation of eosinophils by IL-3 compared to GM-CSF and IL-5. Crit. Rev. Immunol..

[6] Nishinakamura R., Miyajima A., Mee P.J., Tybulewicz V.L., Murray R. (1996). Hematopoiesis in mice lacking the entire granulocyte-macrophage colony-stimulating factor/interleukin-3/interleukin-5 functions. Blood.

[7] Coden M.E., Walker M.T., Jeong B.M., Connelly A.R., Nagasaka R., Berdnikovs S. (2021). Beyond Il-5: Metabolic Reprogramming and Stromal Support Are Prerequisite for Generation and Survival of Long-Lived Eosinophil. Cells.

[8] Son K., Hussain A., Sehmi R., Janssen L. (2021). The Cycling of Intracellular Calcium Released in Response to Fluid Shear Stress Is Critical for Migration-Associated Actin Reorganization in Eosinophils. Cells.

[9] Shen Z.J., Hu J., O’Neal M.A., Malter J.S. (2021). Pin1 Regulates IL-5 Induced Eosinophil Polarization and Migration. Cells.

[10] Shen Z.J., Esnault S., Malter J.S. (2005). The peptidyl-prolyl isomerase Pin1 regulates the stability of granulocyte-macrophage colony-stimulating factor mRNA in activated eosinophils. Nat. Immunol..

[11] Shen Z.J., Esnault S., Schinzel A., Borner C., Malter J.S. (2009). The peptidyl-prolyl isomerase Pin1 facilitates cytokine-induced survival of eosinophils by suppressing Bax activation. Nat. Immunol..

[12] Germic N., Fettrelet T., Stojkov D., Hosseini A., Horn M.P., Karaulov A., Simon D., Yousefi S., Simon H.U. (2021). The Release Kinetics of Eosinophil Peroxidase and Mitochondrial DNA Is Different in Association with Eosinophil Extracellular Trap Formation. Cells.

[13] Bernau K., Leet J.P., Floerke H., Bruhn E.M., Noll A.L., McDermott I.S., Esnault S., Jarjour N.N., Sandbo N. (2021). Interleukin-1alpha Is a Critical Mediator of the Response of Human Bronchial Fibroblasts to Eosinophilic Inflammation. Cells.

[14] Tiwary M., Rooney R.J., Liedmann S., LeMessurier K.S., Samarasinghe A.E. (2021). Eosinophil Responses at the Airway Epithelial Barrier during the Early Phase of Influenza A Virus Infection in C57BL/6 Mice. Cells.

[15] Koenderman L., Siemers M.J., van Aalst C., Bongers S.H., Spijkerman R., Bindels B.J.J., Giustarini G., van Goor H.M.R., Kaasjager K.A.H., Vrisekoop N. (2021). The Systemic Immune Response in COVID-19 Is Associated with a Shift to Formyl-Peptide Unresponsive Eosinophils. Cells.

[16] Salter B.M., Ju X., Sehmi R. (2021). Eosinophil Lineage-Committed Progenitors as a Therapeutic Target for Asthma. Cells.

[17] Cusack R.P., Whetstone C.E., Xie Y., Ranjbar M., Gauvreau G.M. (2021). Regulation of Eosinophilia in Asthma—New Therapeutic Approaches for Asthma Treatment. Cells.

[18] Youngblood B.A., Leung J., Falahati R., Williams J., Schanin J., Brock E.C., Singh B., Chang A.T., O’Sullivan J.A., Schleimer R.P. (2020). Discovery, Function, and Therapeutic Targeting of Siglec-8. Cells.

[19] Masterson J.C., Menard-Katcher C., Larsen L.D., Furuta G.T., Spencer L.A. (2021). Heterogeneity of Intestinal Tissue Eosinophils: Potential Considerations for Next-Generation Eosinophil-Targeting Strategies. Cells.

[20] Iype J., Fux M. (2021). Basophils Orchestrating Eosinophils’ Chemotaxis and Function in Allergic Inflammation. Cells.

